# On Neuralgia

**Published:** 1868-08

**Authors:** 


### SELECTED ARTICLES.

### ARTICLE VI.

### ON NEURALGIA.

By Prof. Trousseau.

In his lecture on neuralgia, Professor Trousseau brings forward his important observation that in all cases of this disease there is more or less accute pain on pressure over the spinous process of those vertebræ which correspond to the origin or point of exit of the affected nerves; and to this *spinous* point he justly attaches very considerable diagnostic value. He draws attention to another peculiarity, concernwhich, he thinks, writers have not been sufficiently explicit —the existence, namely, of cutaneous hyperæsthesia at the point of exit of thə nerve-trunks. This he terms the *spot of peripheral expansion.* He denies the statement of Valleix regarding the *superficial tender spots* to be found in intercostal neuralgia, but admits the existence (amongst others) of those which he indicated in cases where the cranial nerves are affected. Where the neuralgia is superficial, Trousseau finds that the local application of atropine or belladonna is sufficient to relieve pain in the majority of cases. He generally employs a compress steeped in a solution of sulphate of atropine (five grains of sulphate of atropine in three ounces of distilled water), and covered with a piece of oiled silk to prevent evaporation. This application is continued for an hour at a time, and is frequently renewed, provided no disagreeable constitutional effects are produced.

When the neuralgia is more deep-seated or severe, he has recourse to the endermic use of morphia, or the subcutaneous injection of morphia or atropine; and in obstinate cases he makes an incision through the skin, and places in the wound one or two medicated boluses. When he adopts the endermic, method, he removes the cuticle by means of ammonia, as it can be done in this way much more neatly and expeditiously than by means of cantharides. He also advocates the inhalation of chloroform during the attacks.—*Lancet.*

